# Respiratory failure in a patient with dermatomyositis

**DOI:** 10.1186/2049-6958-8-27

**Published:** 2013-03-27

**Authors:** Ivano Salimbene, Ilaria Leli, Salvatore Valente

**Affiliations:** 1 Department of Pulmonary Medicine, A. Gemelli University Polyclinic, Sacro Cuore Catholic University, Largo A. Gemelli 8, Rome, 00168, Italy

**Keywords:** Dermatomyositis, Interstitial disease, Pulmonary complications

## Abstract

Since its original description in 1956 the association between interstitial lung disease and polymyositis (PM) and dermatomyositis (DM) has become well established. Interstitial lung disease (ILD) can be a significant complication in rheumatic diseases (RDs). Although most patients with RD do not develop clinically evident ILD, these systemic autoimmune disorders are estimated to be responsible for approximately 25% of all ILD deaths and 2% of deaths due to all respiratory causes. Radiologic abnormalities in DM are characterized by a high incidence of airspace consolidation. Non-Specific Interstitial Pneumonia (NSIP) is the most common form of lung disease, with a frequency in biopsies 4-fold greater than that of Usual Interstitial Pneumonia (UIP) in PM and a slightly smaller predominance in DM.

We report a case of a female patient, 57 years old, no former smoker, whose clinical history was onset in November 2008 with asthenia with muscle and osteoarticular pain especially located in the upper limbs and then also expanded to the lower limbs. The EMG was compatible with dermatomyositis in the acute phase. The patient received therapy with steroids and tacrolimus, also making several rounds of treatment with immunoglobulin. Given the recurrence of myositis in association with signs of poorly controlled interstitial lung disease, immunosuppressive therapy with Rituximab was administered. The Computed Tomography (CT) scans showed "bronchiectasis and traction bronchiolectasis, hypodense areas consistent with the phenomena of air trapping. The pattern of interstitial lung disease with fibrotic evolution seems consistent with NSIP.

The arterial blood gas analysis showed a pattern of hypoxic-hypercapnic respiratory failure (pH: 7,34, PaO_2_: 67 mmHg; PaCO_2_: 55 mmHg).

As a result of an episode of marked desaturation unresponsive to supplemental oxygen at high flows we proceeded to noninvasive mechanical ventilation with Helmet for 24 hours/24. This ventilatory support was maintained for a week, with resolution of the respiratory failure.

In this brief case report we want to highlight various pulmonary complications as a result of dermatomyositis. The progression of respiratory complications may also lead to a situation of respiratory failure, as in our patient, and require a noninvasive ventilatory treatment.

## Background

Since its original description in 1956 [1], the association between interstitial lung disease and polymyositis (PM) and dermatomyositis (DM) has become well established [2]. Interstitial lung disease (ILD) can be a significant complication in rheumatic diseases (RDs). Although most patients with RD do not develop clinically evident ILD, these systemic autoimmune disorders are estimated to be responsible for approximately 25% of all ILD deaths and 2% of deaths due to all respiratory causes [1]. A particular diagnostic dilemma arises when the onset of lung disease precedes joint, muscle, or skin involvement, or occurs before serologic markers become diagnostic, a process which in some cases takes a few years [3,4]. In patients who do not meet the criteria of the American College of Rheumatologists for the diagnosis of RD, the pathologist may be the first to raise this possibility as a potentially treatable cause of pulmonary disease [5,6]. Through the study of the thoracic pathology of well characterized RD patient populations, we have recognized that each RD has a reasonably characteristic set of acute, subacute, and chronic pleuropulmonary manifestations [7]. Given a limited repertoire of lung repair following injury, the inflammatory and reparative reactions associated with pleuropulmonary RD can cause considerable clinical, radiologic, and histopathologic overlap.

Radiologic abnormalities in DM are characterized by a high incidence of airspace consolidation and a low incidence of honeycombing [8]. Ikezoe et al. [8] have described ground-glass opacities and linear opacities in 92%, irregular interfaces in 88%, air-space consolidation in 52%, parenchymal micronodules in 28%, and honeycombing in 16% of patients. Some radiographic abnormalities, including consolidation and peribronchovascular thickening, can improve with treatment [9]. The most dramatic radiologic finding associated with DM is the development of rapid-onset airspace consolidation, which correlates with acute clinical presentation and acute lung injury patterns in subsequent biopsies [4,10]. Lung involvement is the most common extramuscular manifestation of idiopathic inflammatory myopathies. These patients are traditionally subclassified on the basis of their clinical phenotype, such as PM and DM. Among these groups, NSIP is the most common form of lung disease [3,10,11], with a frequency in biopsies 4-fold greater than that of UIP in PM and a slightly smaller predominance in DM [12-14]. The NSIP is indistinguishable from the idiopathic variant of NSIP, although, when additional features like follicular bronchiolitis are present, the possibility of an underlying RD should be suggested at the time of sign-out . When the fibrosis is more extended, it can often be separated from idiopathic UIP by the lack of centrilobular sparing. About half cases of fibrosing ILD show superimposed BOOP (bronchiolitis obliterans organizing pneumonia), which in some cases is the first manifestation [15-18]. Recently, the discovery of myositis-specific antibodies has prompted stratification of patients into distinct clinical subsets. Antibodies against aminoacyl-transfer RNA synthetases (anti-synthetase antibodies, including Jo-1, PL-7, PL-12, EJ, OJ, and KS) are highly associated with ILD [19]. The most common anti-synthetase antibody Jo-1, found in approximately 20% of patients with myositis, exhibits a greater frequency of UIP than NSIP [20]. Many of these patients present with rapidly progressive hypoxemia and show superimposed acute lung-injury patterns. Pulmonary capillaritis and pulmonary hypertension have rarely been reported in PM/DM [21,22]. Pleuritis, bronchiolitis, and vascular changes are distinctly uncommon in PM/DM and should prompt a search for other possible etiologies. Patients with DM may be at increased risk of developing malignancies [23-26].

The experimental research reported in the manuscript has been performed with the approval of the ethics committee, in compliance with the Helsinki Declaration. The name of the body which gave approval is: Prof. Antonio G. Spagnolo, Sacro Cuore Catholic University, Bioethics Institute, Rome (Italy).

## Case presentation

We report a case of a 57 years old, non smoker woman whose clinical history began in November 2008 with asthenia, malaise with muscle and osteoarticular pain especially located in the upper limbs and then also expanded to the lower limbs. For this reason, she began therapy with methylprednisolone, suspended in January 2009 for an episode of bronchopneumonia and for the remission of muscle symptoms. The following month, because of the worsening of symptoms, like rise of temperature, leg pain and weakness, and asthenia, she was hospitalized. Her following tests were positive: CPK 3683 U/l and anti-Jo1 antibodies 237 U/ml.

**Figure 1:**
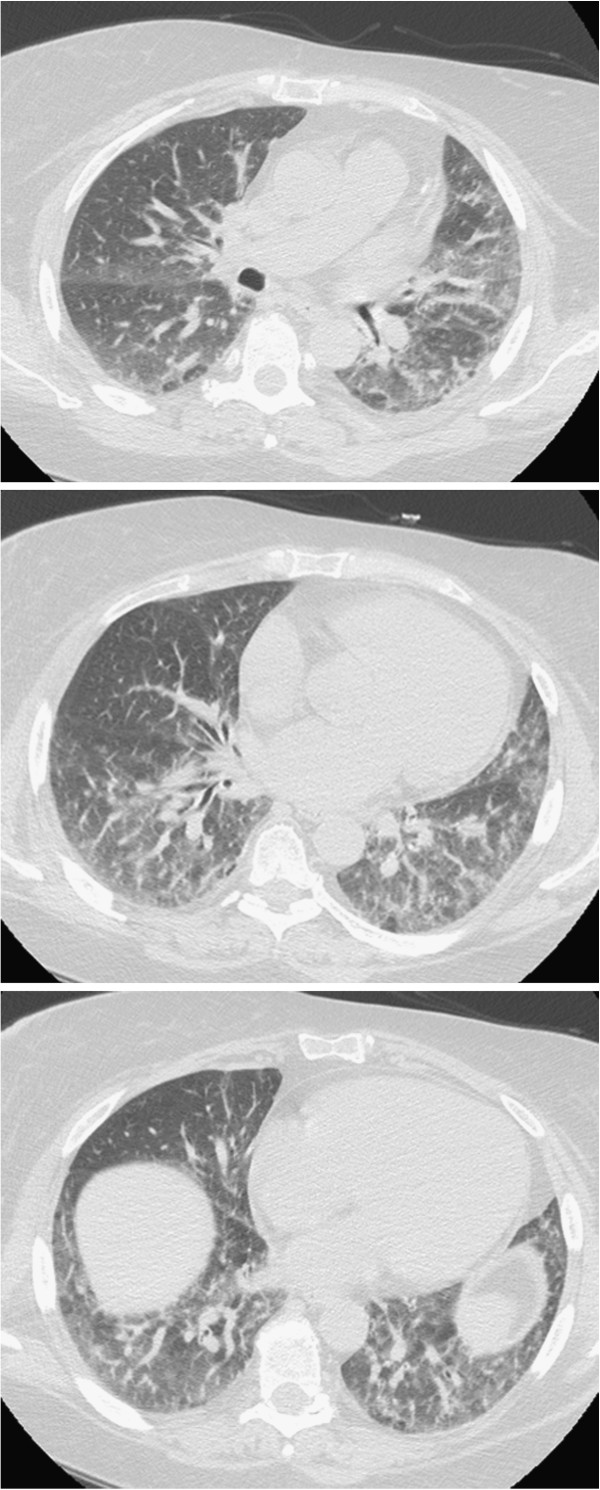
Interstitial disease in phase of activity.

**Figure 2:**
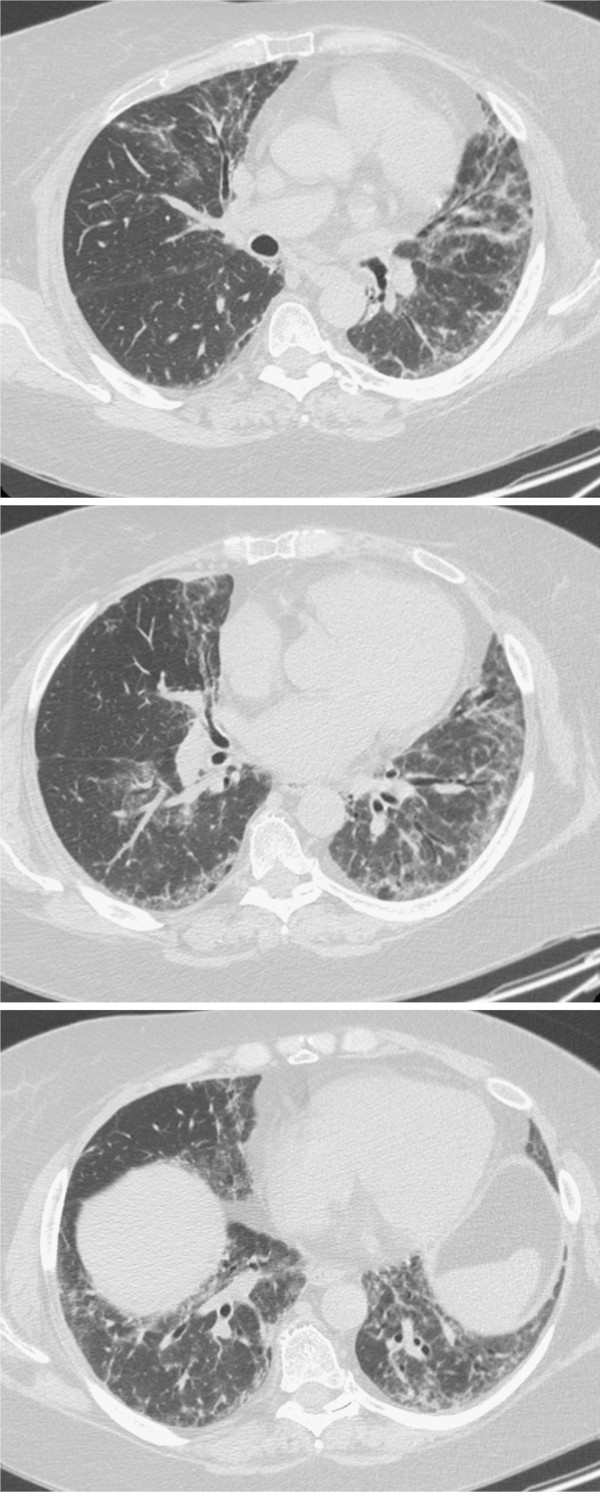
Reduction of the interstitial areas.

The chest CT scan showed: "Minimal pleural thickening, slight signs of pericarditis". Electromyography (EMG) was then performed: "Signs of denervation at the level of some muscles of the lower limbs with sub interferential track at medium-low effort, with motor unit potentials of low amplitude with the presence of polybasic potential. EMG compatible with dermatomyositis in the acute phase". For this reason, she underwent a muscle biopsy which showed "large areas of necrosis and degeneration of the fibers with almost exclusively perifascicular layout and associated diffuse aspects of phagocytosis. Rare inflammatory infiltrates consisting of CD8 + and focal expression of histocompatibility antigens of type I. Morphological pattern of inflammatory myopathy dermatomyositis-like with prevailing aspects of necrosis and phagocytosis. Recommended evaluation to exclude neoplastic diseases associated with". She also performed a skin biopsy that showed "atrophic epidermis with hyperkeratosis. The papillary dermis is edematous, with vascular ectasis and a moderate lymphoplasmacytic inflammatory infiltrate predominantly perivascular". Since then she took prednisone 75 mg/day with a gradual reduction to 25 mg/day until July 2009. The following month she was admitted to the hospital and she was subjected again to biopsy of the deltoid muscle with evidence of a typical pattern of dermatomyositis. She began therapy with cyclosporine, well tolerated and with benefit. The thoracic HRCT scans performed in 2010 (Figure 1) showed "interstitial disease in phase of activity". From June 2010 she suspended cyclosporine and began immunosuppressive treatment with tacrolimus. The control CT scans in September 2010 (Figure 2) gave a reduction of the interstitial areas. Then she performed several rounds of immunoglobulin treatment, continuing treatment with tacrolimus and steroids.

Given the recurrence of myositis with association of interstitial lung disease poorly controlled by immunosuppressive therapy (tacrolimus, cyclosporine) and considering the documented efficacy of immunosuppressive therapy with rituximab in the treatment of resistant forms of dermatomyositis with anti-Jo1 antibodies, the administration of this drug during hospitalization (after permission from the Ethic Committee) was proposed to the patient and she did not present any adverse effects.

In the following controls a reduction in muscle CK and a persistent reduction in the B-lymphocyte population was detected, as a result of the immunosuppressive therapy. In September 2011, as a result of left leg pain, a venous Doppler ultrasound examination of the inferior limbs was performed and showed "to the left, thrombosis of the tibio-peroneal trunk and of the popliteal artery". Low molecular weight heparin was performed (6000UI X 2/day). In November 2012 the patient was hospitalized because of fever (maximum 38°C), with continuous-remitting course, accompanied by increase in muscle enzymes, recurrence of muscle pain and weakness with involvement of the pelvic and scapular girdle and for the presence of haematic sputum. The blood count was normal except for a slight increase in white blood cells (10.50* 10^9/l), mild hypokalemia (3,4 mEq/l) and hypercholesterolemia (204 mg/dl). The erythrocyte sedimentation rate was slightly increased (37 mm), as the dosage of creatine kinase (CK) was: 2649 UI/l.

**Figure 3:**
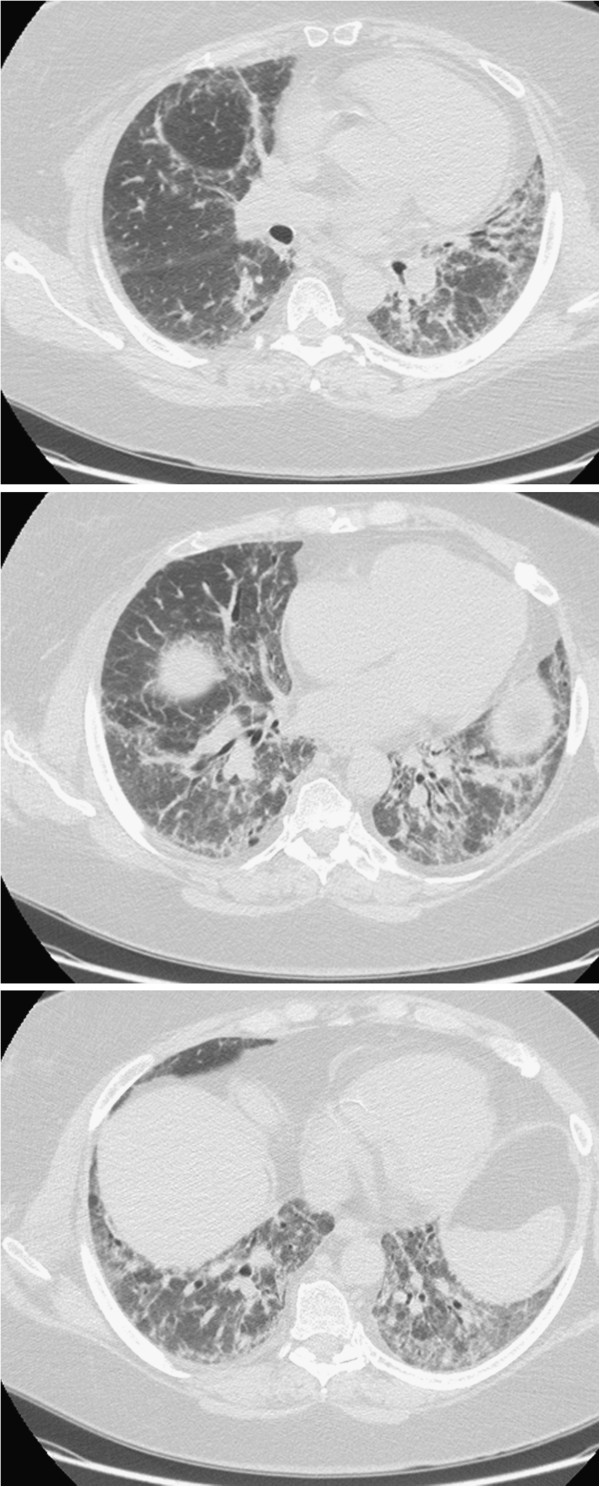
**Increased areas of increased density with ground-glass appearance, involving the lower lobes and the left upper lobe.** Bronchiectasis and traction bronchiolectasis. Hypodense areas consistent with air trapping phenomena. The pattern of interstitial lung disease with fibrotic evolution seems at first hypothesis consistent with NSIP.

Chest X-ray was performed and showed "an area of alveolar involvement that overlaps to the chronic bilateral interstitial lung disease pattern, relating to an acute inflammatory process and/or to a worsening of underlying pulmonary fibrosis, in the absence of pleural effusion". So an HRCT was performed (Figure 3) that showed, compared with the previous, “increased areas of increased density with ground-glass appearance, involving the lower lobes and the LUL (left upper lobe). Bronchiectasis and traction bronchiolectasis. Hypodense areas consistent with air trapping phenomena. The pattern of interstitial lung disease with fibrotic evolution seems consistent with NSIP, in the first hypothesis”.

An arterial blood gas analysis was performed (FiO_2_ 0,21) and showed PaO_2_ 57 mmHg, PaCO_2_: 33,7 mmHg, pH 7,47, HCO3-: 25,6 mEq/l. The three samples of the bacterial culture of sputum were found to be positive for Staphylococcus Aureus, Serratia Marcescens and Citrobacter Freundii and they did not show a predominance of a species. So the patient began oxygen therapy with Venturi mask with FiO_2_ 0,28 for 24 h (using nasal cannulas with a flow rate of 2 lpm only during meals) and antibiotic therapy with ciprofloxacin 400 mg/iv x 2/day and Ceftazidime 2 g/ev x 3/die. As a result of a marked desaturation episode unresponsive to supplemental oxygen at high flows she was submitted to a non-invasive mechanical ventilation, with Helmet, 24 h/24. This support was maintained for a week, with improvement of the respiratory failure.

## Discussion

This brief case report wants to put in evidence how many pulmonary complications can be the result of dermatomyositis. The patient developed a pulmonary infection by Staphylococcus aureus, Serratiamarcescens and Citrobacterfreundii; patients in the steroid treatment are more susceptible to infections. In addition, the typical pattern of interstitial lung disease NSIP-like is very common in patients with dermatomyositis.

Other pulmonary complications in patients with dermatomyositis may be aspiration pneumonia secondary to dysphagia, ventilatory failure secondary to muscular weakness, primary or metastatic malignancy, pleural effusion, spontaneous pneumothorax.

The progression of respiratory complications may also lead to a situation of respiratory failure as in the case of our patient and requires a non-invasive ventilatory treatment.

The five rheumatic diseases most frequently associated with pleuropulmonary disease are rheumatoid arthritis, systemic lupus erythematosus, progressive systemic sclerosis, polymyositis/dermatomyositis, and Sjögren syndrome. The onset of thoracic involvement in these diseases is variable. In some patients, it precedes the systemic disease or is its only manifestation. Moreover, there is a wide spectrum of clinical presentation ranging from subclinical abnormalities to acute respiratory failure. Histopathologically, the hallmark features of thoracic involvement by RD are inflammatory, targeting one or more lung compartments. The reactions range from acute to chronic, with remodeling by fibrosis being a common result. Although the inflammatory findings are often nonspecific, certain reactions or anatomic distributions may favor one RD over another, and occasionally, a distinctive histopathology may be present (e.g., rheumatoid nodules). Three diagnostic dilemmas are encountered in patients with RD who develop diffuse lung disease: 1) opportunistic infection in the immunocompromised host, 2) drug toxicity related to the medications used to treat the systemic disease, and 3) manifestations of the patient's known systemic disease in lung and pleura [27].

## Conclusions

The most common patterns of interstitial pneumonia in polymyositis and dermatomyositis are nonspecific interstitial pneumonia and organizing pneumonia [28]. The two patterns are not mutually exclusive; both may be seen in lung biopsy specimens from the same patient. Organizing pneumonia may occur either as a primary manifestation of polymyositis or dermatomyositis or may result from treatment of these systemic diseases with azathioprine, methotrexate, cyclophosphamide, or cyclosporine. Furthermore, dermatomyositis is associated with an increase in the incidence of malignancies of the cervix, lung, pancreas, breast, ovaries, and gastrointestinal tract, particularly among patients older than 60 years [29].

## Consent

Written informed consent was obtained from the patient for publication of this report and any accompanying images.

## Competing interest

The authors declared that they have no competing interest.
